# Molecular Dynamics Simulation and MM/PBSA Analysis of α-Mangostin Stabilization in Soluplus^®^ and Kollidon^®^ VA64-Based Amorphous Solid Dispersions

**DOI:** 10.3390/ijms27167283

**Published:** 2026-08-15

**Authors:** Ferdy Firmansyah, Arif Budiman, Muchtaridi Muchtaridi, Taufik Muhammad Fakih, Ahmed Fouad Abdelwahab Mohammed, Safwat A. Mahmoud, Khaled M. Elamin, Nasrul Wathoni

**Affiliations:** 1 Doctoral Program of Pharmacy, Faculty of Pharmacy, Universitas Padjadjaran, Jatinangor 45363, West Java, Indonesia; ferdy24001@mail.unpad.ac.id; 2 Department of Pharmacy, Sekolah Tinggi Ilmu Farmasi Riau, Pekanbaru 28289, Riau, Indonesia; 3 Department of Pharmaceutics and Pharmaceutical Technology, Faculty of Pharmacy, Universitas Padjadjaran, Jatinangor 45363, West Java, Indonesia; arif.budiman@unpad.ac.id; 4 Department of Pharmaceutical Analysis and Medicinal Chemistry, Faculty of Pharmacy, Universitas Padjadjaran, Jatinangor 45363, West Java, Indonesia; 5 Research Collaboration Centre for Radiopharmaceuticals Theranostics, National Research and Innovation Agency (BRIN), Jatinangor 45363, West Java, Indonesia; 6 Department of Pharmacy, Faculty of Mathematics and Natural Sciences, Universitas Islam Bandung, Bandung 40166, West Java, Indonesia; 7 Department of Pharmaceutics, Faculty of Pharmacy, Minia University, Minia 61519, Egypt; ahmed.mohamed1@minia.edu.eg; 8 Department of Pharmaceutics, Faculty of Pharmacy, Minia National University, Minia 61768, Egypt; 9 Center for Scientific Research and Entrepreneurship, Northern Border University, Arar 73213, Saudi Arabia; safwat.abdulrab@nbu.edu.sa; 10 Graduate School of Pharmaceutical Sciences, Kumamoto University, Kumamoto 860-8555, Japan; khaled@kumamoto-u.ac.jp

**Keywords:** α-Mangostin, amorphous solid dispersion, molecular dynamics, MM/PBSA, Soluplus^®^, Kollidon^®^ VA64, drug–polymer interactions

## Abstract

α-Mangostin (αM) is a natural xanthone with broad pharmacological activity; however, its therapeutic use is limited by poor aqueous solubility and low bioavailability. Amorphous Solid Dispersion (ASD) is a practical strategy for improving poorly soluble drugs; however, polymer selection remains a critical step that should be supported by molecular-level evidence of drug–polymer compatibility. In this study, 500 ns Molecular Dynamics (MD) simulations followed by Molecular Mechanics/Poisson–Boltzmann Surface Area (MM/PBSA) analysis were used to compare the association of αM with Soluplus^®^ and Kollidon^®^ VA64 at drug-to-polymer ratios of 1:1, 1:3, 1:5, and 1:7. All systems reached relatively stable configurations, but the two polymers stabilized αM differently. Soluplus^®^ 1:3 produced the most stable global structure based on RMSD, whereas Kollidon^®^ VA64 showed its best stability at 1:7. Soluplus^®^ displayed a stronger interaction network: at 1:7, it generated 1150 hydrogen-bond pairs with 231.17% cumulative occupancy, compared with 900 pairs and 96.69% for Kollidon^®^ VA64. Binding energies ranged from −126.24 to −484.09 kJ/mol for Soluplus^®^ and from −71.58 to −382.42 kJ/mol for Kollidon^®^ VA64, mainly driven by van der Waals contacts. These results indicate that Soluplus^®^ provides a more favorable molecular environment for further αM ASD development.

## 1. Introduction

α-Mangostin (αM) is a prenylated xanthone primarily isolated from the pericarp of *Garcinia mangostana* and has demonstrated antimicrobial, antibiofilm, anticancer, antioxidant, and anti-inflammatory activities [1,2,3,4,5,6,7]. However, its pharmaceutical development is constrained by its high hydrophobicity, poor aqueous solubility, limited dissolution in physiological media, and strong tendency to remain in a thermodynamically stable crystalline state [8,9]. These properties reduce the fraction available for dissolution and biological interaction and may contribute to poor in vivo performance. Conventional formulations may also be unable to maintain αM in a sufficiently dispersed state because of aggregation, phase separation, or recrystallization [10]. Nanoparticles, nanomicelles, lipid-based carriers, and solid dispersions have therefore been investigated to improve its apparent solubility and bioavailability [11,12,13,14]. Nevertheless, dissolution enhancement alone is insufficient unless the drug can also be maintained in a molecularly dispersed and physically stable form.

Amorphous solid dispersion (ASD) is a relevant strategy because it reduces the crystal lattice barrier by dispersing the drug within a polymeric matrix in a partially or fully amorphous state [15,16,17]. This higher-energy form may improve apparent solubility and dissolution; however, it is thermodynamically metastable and susceptible to molecular relaxation, phase separation, nucleation, and recrystallization during storage or exposure to aqueous media [15,18,19]. Hydration, polymer mobility, and local drug concentrations may further disrupt the initial homogeneity of the dispersion. Accordingly, ASD performance depends not only on achieving amorphization, but also on preserving the amorphous state over a relevant period.

The polymer plays a central role in restricting molecular mobility, limiting drug-rich domain formation, and delaying crystal nucleation and growth [18,19,20,21]. Stabilization is governed by drug–polymer compatibility and the balance among hydrogen bonding, hydrophobic interactions, van der Waals forces, and electrostatic contributions [18,19,22]. Persistent drug–polymer interactions may suppress drug reassociation, whereas dominant drug–drug interactions can promote clustering and the development of nucleation-prone domains [20,21,23]. Inadequate compatibility may therefore result in phase separation, recrystallization, and loss of the initial solubility advantage [19,23,24].

Soluplus^®^ and Kollidon^®^ VA64 are well-established polymeric carriers in ASD development because their distinct chemical structures can stabilize hydrophobic drugs [23,24]. Soluplus^®^ is an amphiphilic graft copolymer containing hydrophilic and hydrophobic segments that can interact with poorly soluble drugs through both polar and non-polar contacts. Kollidon^®^ VA64, or copovidone, contains vinylpyrrolidone and vinyl acetate moieties, including lactam groups that can act as hydrogen-bond acceptors [25,26]. Differences in amphiphilicity, polarity, chain flexibility, and interaction capacity may therefore lead to different stabilization behavior, even at comparable drug-to-polymer ratios [27,28].

The experimental characterization of ASD systems provides important information on crystallinity, thermal transitions, and dissolution performance [23,24]. Molecular dynamics (MD) simulation offers an atomistic approach for examining molecular mobility, packing, solvent exposure, preferential interaction distances, and energetic contributions within ASD systems [29,30,31,32,33]. Global and local dynamics can be assessed using root mean square deviation (RMSD) and root mean square fluctuation (RMSF), while radius of gyration (Rg) and solvent-accessible surface area (SASA) provide complementary information on compactness and solvent exposure [33,34,35]. Radial distribution function analysis can identify preferential distances and spatial associations between drug and polymer functional groups [31,36,37]. Hydrogen bonding, interaction-energy calculations, and molecular mechanics/Poisson–Boltzmann surface area analysis further characterize the non-covalent and energetic basis of molecular affinity [38,39,40,41]. Together, these descriptors provide a multidimensional assessment of structural organization and drug–polymer stabilization.

**Figure 1:**
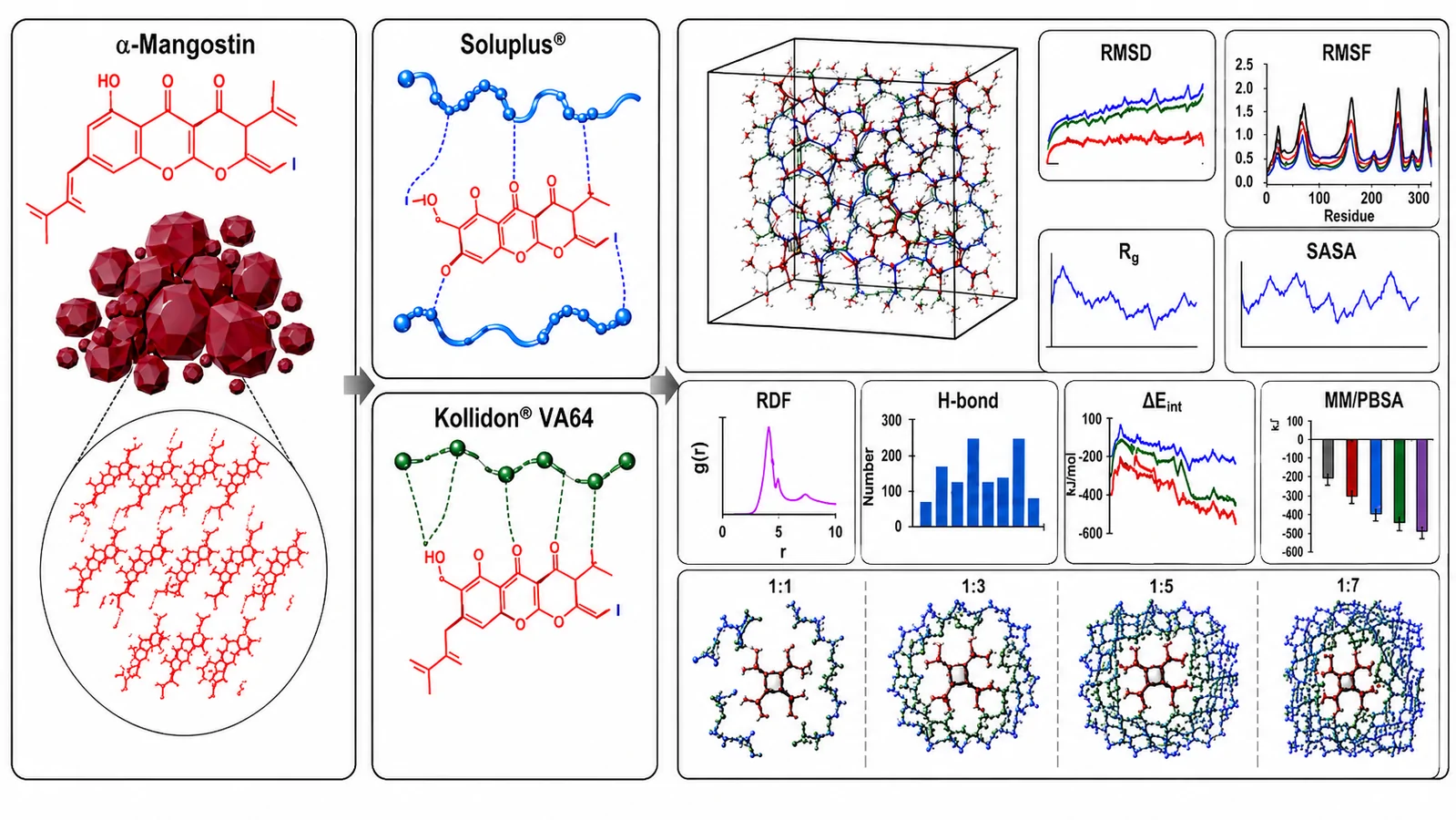
Conceptual framework of the molecular dynamics-guided evaluation of αM ASD stabilized by Soluplus^®^ and Kollidon^®^ VA64.

As illustrated in Figure 1, this study evaluated the molecular stabilization of αM in Soluplus^®^- and Kollidon^®^ VA64-based ASD systems across different drug-to-polymer ratios. The analysis integrates structural, spatial, and energetic descriptors to determine how the polymer type and composition influence the molecular organization, interaction persistence, and relative stability of the simulated dispersions.

Although ASD has been explored to improve the performance of αM and other hydrophobic compounds, the atomistic understanding of how Soluplus^®^ and Kollidon^®^ VA64 stabilize αM at different drug-to-polymer ratios remains limited. Previous studies on αM-based ASD have mainly emphasized experimental characterization, dissolution improvement, or the use of selected polymeric carriers [14,29,36]. Our previous molecular dynamics study focused on αM interactions with poloxamer and pullulan, which possess physicochemical characteristics that differ substantially from Soluplus^®^ and Kollidon^®^ VA64. Consequently, the molecular stabilization mechanisms identified in those systems cannot be directly extrapolated to the present polymer matrices because differences in polymer architecture, amphiphilicity, and functional groups may lead to distinct drug–polymer interaction patterns. However, the relationship between polymer type, drug ratio, dynamic stability, molecular compactness, solvent exposure, spatial distribution of functional groups, hydrogen bond formation, and binding energy has not been systematically clarified. This gap is important because two polymers may produce apparently similar macroscopic outcomes while stabilizing the drug through different molecular pathways. Therefore, this study aimed to compare the molecular stabilization mechanisms of αM by Soluplus^®^ and Kollidon^®^ VA64 at drug-to-polymer ratios of 1:1, 1:3, 1:5, and 1:7 using MD simulation and MM/PBSA analysis. Unlike our previous work, the objective of this study was not merely to evaluate different polymeric carriers but to elucidate how two pharmaceutically relevant ASD polymers with distinct molecular architectures govern the stabilization of αM over a broad range of drug-to-polymer ratios. This design enables the systematic evaluation of the interplay between polymer chemistry, polymer concentration, and molecular stabilization mechanisms, thereby providing mechanistic insights that extend beyond formulation performance alone. Rather than treating polymer selection as an empirical screening step, this study maps how two chemically distinct polymers stabilize αM across increasing polymer ratios by integrating structural dynamics, molecular compactness, solvent exposure, non-covalent interactions, and binding free energy. These mechanistic insights provide a molecular-level framework for understanding polymer-dependent stabilization in ASD systems and contribute to the rational design of carrier selection for poorly water-soluble compounds, which aligns with the molecular science focus of this study. Previous studies have shown that polymer composition and drug–polymer interactions can substantially influence ASD stability and dissolution behavior; however, this relationship has not been specifically resolved for αM–Soluplus^®^ and αM–Kollidon^®^ VA64 systems at the atomistic level. The findings are expected to provide a rational molecular basis for selecting suitable polymeric carriers and drug-to-polymer ratios before further formulation development of αM. This study contributes to SDG 3 (Target 3.b) and SDG 9 (Target 9.5) by advancing molecularly guided polymer selection for poorly water-soluble drugs.

## 2. Results

### 2.1. RMSD-Based Structural Stability Analysis

**Figure 2:**
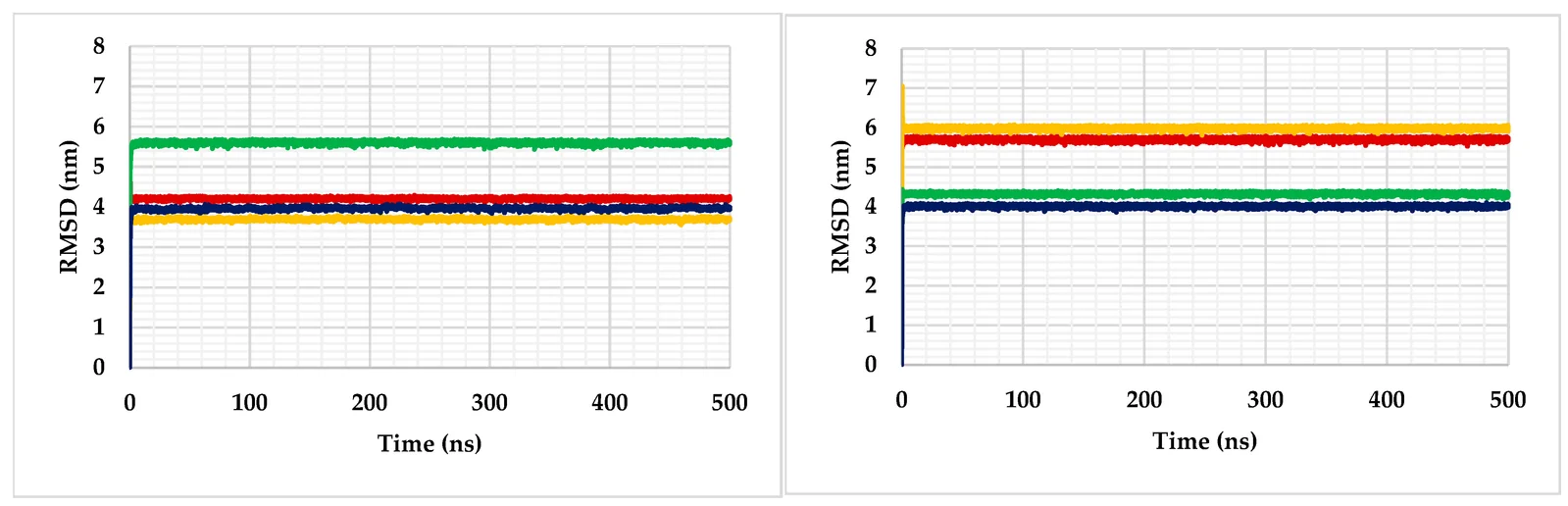
Time-dependent RMSD profiles of αM–polymer systems over 500 ns, illustrating the comparative stability of Soluplus^®^ (**left**) and Kollidon^®^ VA64 (**right**) at varying composition ratios of 1:1 (red), 1:3 (yellow), 1:5 (green), and 1:7 (blue).

The RMSD profiles of the αM–polymer systems during the 500 ns molecular dynamics simulations are shown in Figure 2. Overall, all systems exhibited an initial increase in RMSD, followed by a relatively stable plateau until the end of the trajectory. For quantitative comparison, the 10–500 ns interval was used as the main analysis period, whereas the 400–500 ns interval was used to evaluate the late-trajectory stability.

In the αM–Soluplus^®^ systems, the RMSD varied across the investigated composition ratios. Based on the single 500 ns trajectory generated for each system, the 1:3 composition exhibited the lowest mean RMSD, with values of 3.6968 ± 0.0255 nm over 10–500 ns and 3.6924 ± 0.0273 nm over the final 100 ns, indicating a relatively consistent structural behavior during the late stage of the simulation. The corresponding values for the 1:1 system were 4.2090 ± 0.0239 and 4.2086 ± 0.0234 nm, respectively. The 1:7 system showed mean RMSD values of 3.9650 ± 0.0301 nm over 10–500 ns and 3.9679 ± 0.0277 nm over 400–500 ns. In contrast, the 1:5 system exhibited the highest RMSD, with values of 5.6029 ± 0.0326 and 5.6008 ± 0.0299 nm for the same analysis windows, respectively. The final-frame RMSD values were 4.2055, 3.7037, 5.6058, and 3.9440 nm for the 1:1, 1:3, 1:5, and 1:7 systems, respectively.

In the αM–Kollidon^®^ VA64 systems, the mean RMSD values over 10–500 ns ranged from 4.0120–5.9851 nm. The 1:7 composition exhibited the lowest RMSD, with mean values of 4.0120 ± 0.0274 nm over 10–500 ns and 4.0117 ± 0.0268 nm over the final 100 ns, and a final-frame value of 4.0314 nm. The 1:5 system showed corresponding values of 4.3272 ± 0.0277 and 4.3255 ± 0.0294 nm, with a final RMSD of 4.3378 nm. Higher RMSD values were observed for the 1:1 system, which yielded 5.7030 ± 0.0322 nm over 10–500 ns and 5.7036 ± 0.0335 nm over 400–500 ns, with a final value of 5.7027 nm. The 1:3 composition exhibited the highest RMSD within the Kollidon^®^ VA64 group, with mean values of 5.9851 ± 0.0309 and 5.9865 ± 0.0292 nm for the two analysis windows, respectively, and a final RMSD of 5.9663 nm.

### 2.2. RMSF-Based Structural Flexibility Analysis

**Figure 3:**
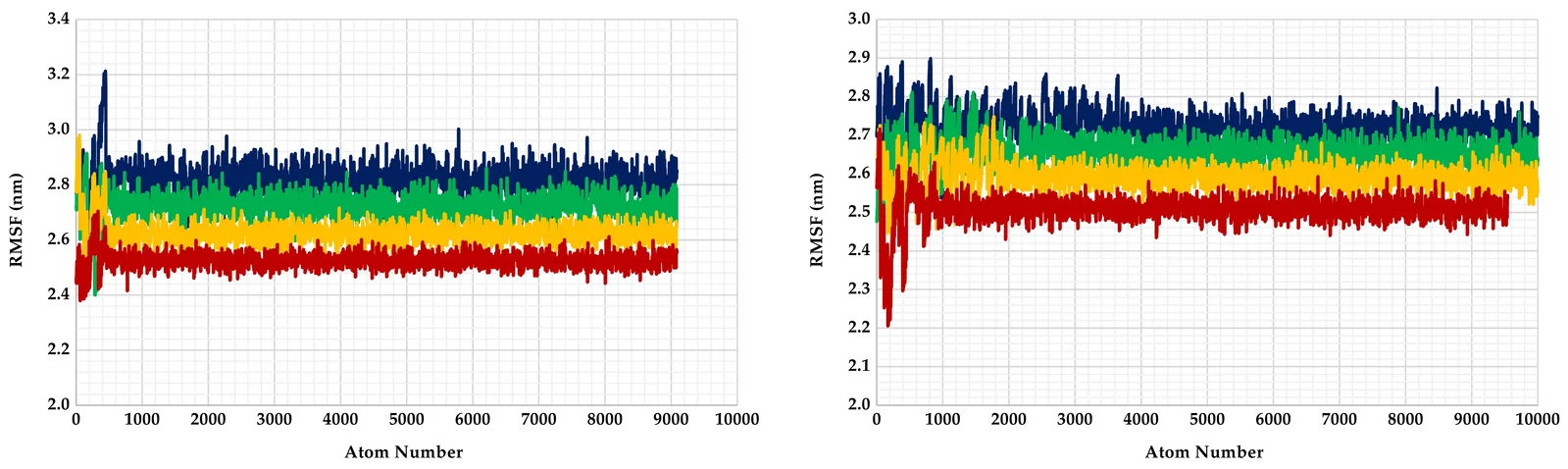
Atomic fluctuation (RMSF) profiles of αM–polymer systems, illustrating the flexibility of Soluplus^®^ (**left**) and Kollidon^®^ VA64 (**right**) at varying composition ratios of 1:1 (red), 1:3 (yellow), 1:5 (green), and 1:7 (blue).

The root mean square fluctuation (RMSF) profiles of the αM–polymer systems are presented in Figure 3. RMSF analysis was used to describe atomistic fluctuations throughout the simulation, where higher values indicate larger positional deviations of the analyzed atoms. Overall, all systems showed relatively consistent RMSF profiles across most of the analyzed atoms, although the magnitude of the fluctuations varied among the polymer types and composition ratios.

In the αM–Soluplus^®^ systems, the mean RMSF values varied across different composition ratios. The αM–Soluplus^®^ 1:1 system showed the lowest mean RMSF within the Soluplus^®^ group, with a value of 2.5264 ± 0.0297 nm and a range of 2.3791–2.7046 nm. The αM–Soluplus^®^ 1:3 system showed a mean RMSF of 2.6253 ± 0.0418 nm, with values ranging from 2.4220–2.9804 nm. The αM–Soluplus^®^ 1:5 system exhibited a mean RMSF of 2.7236 ± 0.0491 nm, with a range of 2.4002–2.9141 nm. The highest mean RMSF in the Soluplus^®^ group was observed in the αM–Soluplus^®^ 1:7 system, with a value of 2.8185 ± 0.0601 nm and a range of 2.6074–3.2128 nm.

In the αM–Kollidon^®^ VA64 systems, the mean RMSF values differed among the composition ratios. The αM–Kollidon^®^ VA64 1:1 system showed a mean RMSF of 2.5078 ± 0.0396 nm, with values ranging from 2.2054–2.7162 nm. The αM–Kollidon^®^ VA64 1:3 system showed a mean RMSF of 2.5917 ± 0.0353 nm, with a range of 2.4269–2.7479 nm. The αM–Kollidon^®^ VA64 1:5 system exhibited a mean RMSF of 2.6555 ± 0.0381 nm, with values ranging from 2.4272–2.8114 nm. The highest mean RMSF within the Kollidon^®^ VA64 group was observed in the αM–Kollidon^®^ VA64 1:7 system, with a value of 2.7195 ± 0.0413 nm (range: 2.5216–2.8990 nm).

### 2.3. RDF-Based Intermolecular Distribution Analysis

**Figure 4:**
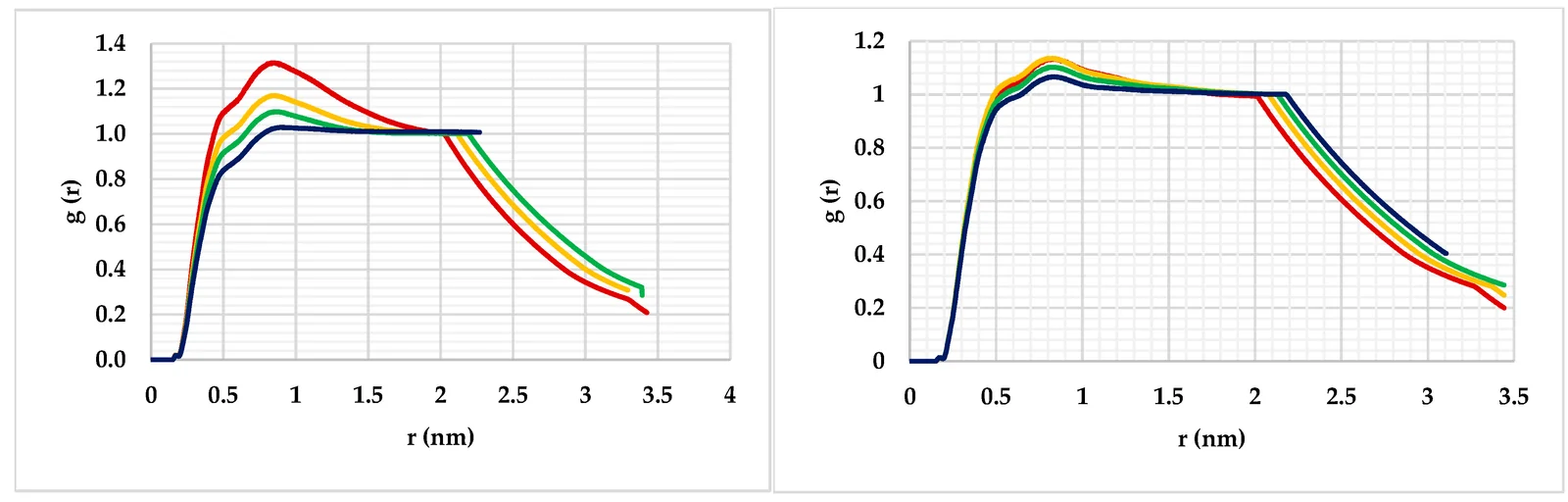
RDF profiles illustrating the intermolecular interaction distances in αM–polymer systems, comparing Soluplus^®^ (**left**) and Kollidon^®^ VA64 (**right**) across the composition ratios of 1:1 (red), 1:3 (yellow), 1:5 (green), and 1:7 (blue).

The radial distribution function (RDF) profiles of the αM–polymer systems are presented in Figure 4. Overall, all systems exhibited an increase in g(r) at short intermolecular distances, followed by a maximum peak within the range of approximately 0.812–0.890 nm, and a gradual decrease at longer distances. Differences in peak intensity and position were observed among the composition ratios, indicating variations in the distance-dependent distribution between αM and the polymer matrices.

In the αM–Soluplus^®^ system, the RDF profiles exhibited distinct peak intensities across the composition ratios. The αM–Soluplus^®^ 1:1 system exhibited the highest RDF peak within this group, with a maximum g(r) value of 1.315 at 0.834 nm. The αM–Soluplus^®^ 1:3 system showed a maximum g(r) value of 1.169 at a distance of 0.834 nm. At the 1:5 ratio, the maximum g(r) decreased to 1.098 at 0.844 nm. The αM–Soluplus^®^ 1:7 system displayed the lowest RDF peak in the Soluplus^®^ group, with a maximum g(r) value of 1.029 at 0.890 nm. Thus, the RDF peak positions in the αM–Soluplus^®^ systems were observed within the range of 0.834–0.890 nm.

In the αM–Kollidon^®^ VA64 systems, the RDF peaks were observed within a relatively narrow distance range of 0.812–0.830 nm. The αM–Kollidon^®^ VA64 1:1 system showed a maximum g(r) value of 1.136 at 0.830 nm, while the 1:3 system showed a comparable peak intensity, with a maximum g(r) value of 1.137 at 0.826 nm. The αM–Kollidon^®^ VA64 1:5 system exhibited a maximum g(r) value of 1.103 at 0.812 nm. Meanwhile, the αM–Kollidon^®^ VA64 1:7 system showed the lowest RDF peak within the Kollidon^®^ VA64 group, with a maximum g(r) value of 1.067 at 0.822 nm.

### 2.4. Rg-Based Structural Compactness Analysis

**Figure 5:**
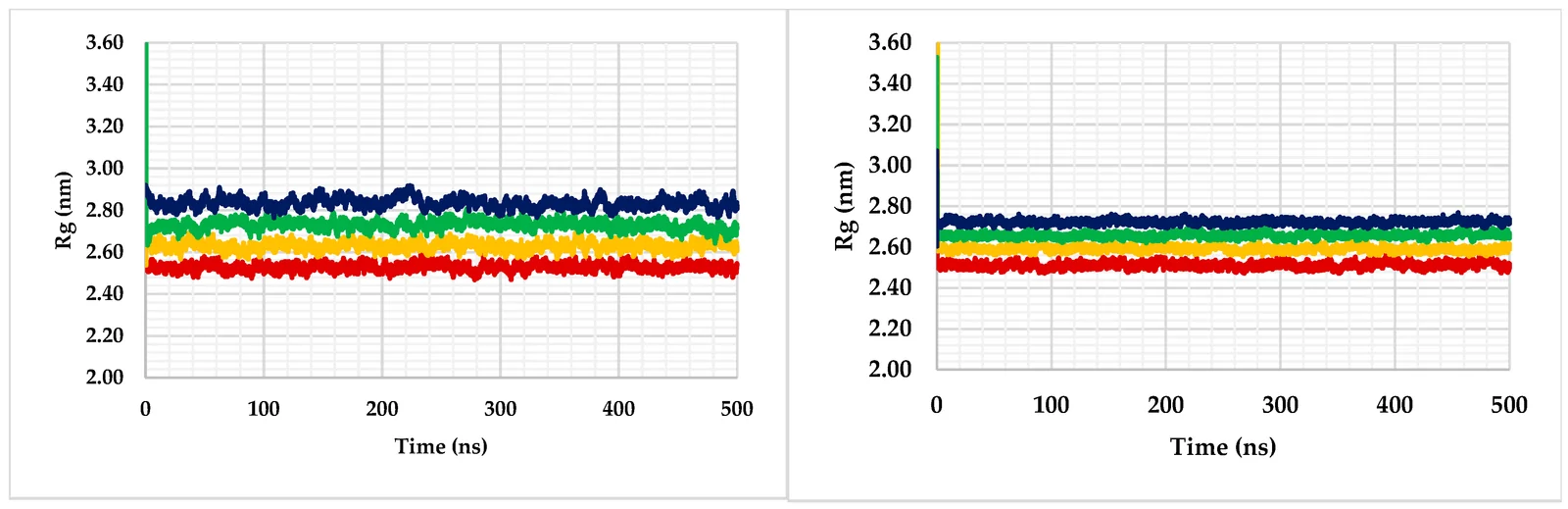
Rg profiles illustrating the structural compactness of αM–polymer systems over 500 ns, comparing Soluplus^®^ (**left**) and Kollidon^®^ VA64 (**right**) across composition ratios of 1:1 (red), 1:3 (yellow), 1:5 (green), and 1:7 (blue).

The radius of gyration (Rg) profiles of the αM–polymer systems during the 500 ns molecular dynamics simulations are shown in Figure 5. Overall, all systems exhibited relatively stable Rg profiles after the initial stage of the simulation. For quantitative comparison, the 10–500 ns interval was used as the main analysis period, whereas the 400–500 ns interval was used to evaluate the late-trajectory compactness.

In the αM–Soluplus^®^ system, the Rg values varied with the composition ratios. The αM–Soluplus^®^ 1:1 system showed a mean Rg of 2.5281 ± 0.0165 nm during 10–500 ns and 2.5287 ± 0.0145 nm during 400–500 ns, with a final Rg value of 2.5415 nm. The αM–Soluplus^®^ 1:3 system showed a mean Rg of 2.6301 ± 0.0198 nm during 10–500 ns and 2.6308 ± 0.0193 nm during 400–500 ns, with a final value of 2.6058 nm. The αM–Soluplus^®^ 1:5 system exhibited a mean Rg of 2.7300 ± 0.0202 nm during 10–500 ns and 2.7173 ± 0.0214 nm during 400–500 ns, with a final value of 2.7159 nm. The αM–Soluplus^®^ 1:7 system showed the highest Rg within the Soluplus^®^ group, with mean values of 2.8334 ± 0.0234 nm and 2.8311 ± 0.0204 nm during 10–500 ns and 400–500 ns, respectively, and a final value of 2.8070 nm.

In the αM–Kollidon^®^ VA64 systems, the Rg values were distributed within a narrower range than those observed in the Soluplus^®^ systems. The αM–Kollidon^®^ VA64 1:1 system showed a mean Rg of 2.5116 ± 0.0133 nm during 10–500 ns and 2.5144 ± 0.0144 nm during 400–500 ns, with a final value of 2.5005 nm. The αM–Kollidon^®^ VA64 1:3 system showed a mean Rg of 2.5915 ± 0.0118 nm during 10–500 ns and 2.5870 ± 0.0095 nm during 400–500 ns, with a final value of 2.5974 nm. The αM–Kollidon^®^ VA64 1:5 system exhibited a mean Rg of 2.6576 ± 0.0110 nm during 10–500 ns and 2.6613 ± 0.0118 nm during 400–500 ns, with a final value of 2.6666 nm. The αM–Kollidon^®^ VA64 1:7 system showed the highest Rg within the Kollidon^®^ VA64 group, with mean values of 2.7226 ± 0.0101 nm and 2.7253 ± 0.0095 nm during 10–500 ns and 400–500 ns, respectively, and a final value of 2.7316 nm.

### 2.5. SASA-Based Surface Exposure Analysis

**Figure 6:**
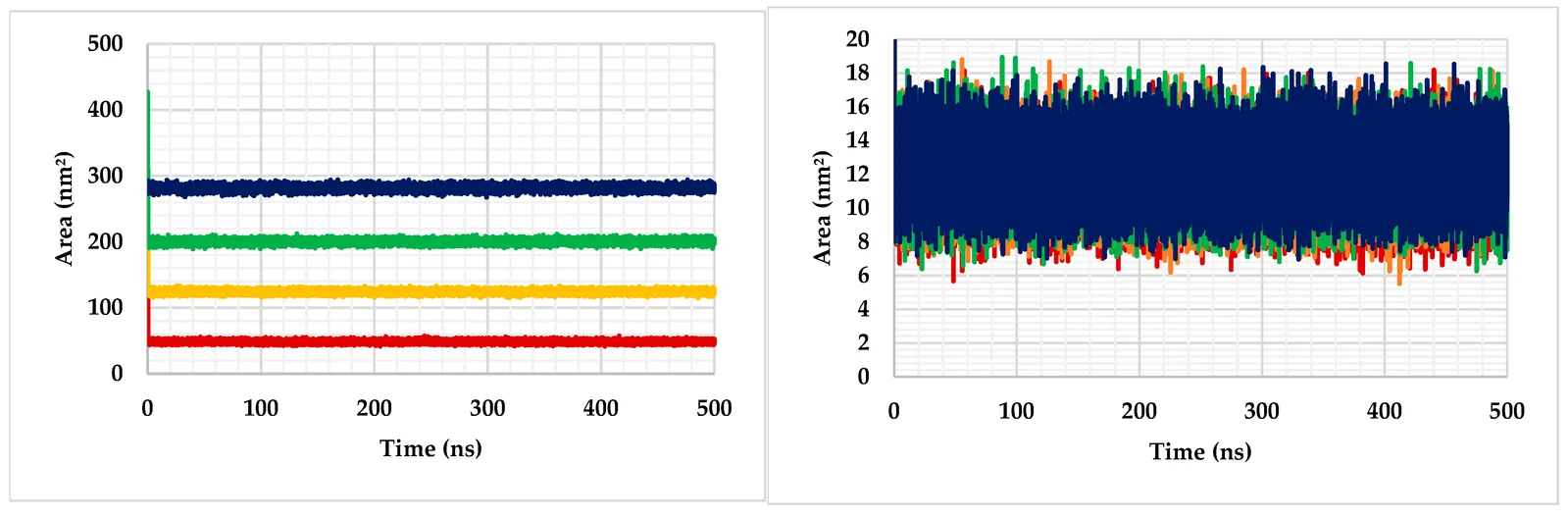
SASA profiles illustrating the molecular surface exposure of αM–polymer systems over 500 ns, comparing Soluplus^®^ (**left**) and Kollidon^®^ VA64 (**right**) across composition ratios of 1:1 (red), 1:3 (yellow), 1:5 (green), and 1:7 (blue).

The solvent-accessible surface area (SASA) profiles of the αM–polymer systems during the 500 ns of molecular dynamics simulations are shown in Figure 6. Overall, the SASA profiles remained relatively stable after the early simulation phase, although the absolute surface areas differed markedly between the Soluplus^®^ and Kollidon^®^ VA64 systems. For quantitative comparison, the 10–500 ns interval was used as the main analysis period, whereas the 400–500 ns interval was used to evaluate the late-trajectory surface exposure.

In the αM–Soluplus^®^ system, the SASA values increased with increasing polymer ratio. The αM–Soluplus^®^ 1:1 system showed a mean SASA of 48.42 ± 1.90 nm^2^ during 10–500 ns and 48.46 ± 1.93 nm^2^ during 400–500 ns, with a final value of 47.51 nm^2^. The αM–Soluplus^®^ 1:3 system exhibited a higher mean SASA of 124.18 ± 2.39 nm^2^ during 10–500 ns and 124.24 ± 2.38 nm^2^ during 400–500 ns, with a final value of 128.70 nm^2^. The αM–Soluplus^®^ 1:5 system showed a mean SASA of 200.19 ± 2.84 nm^2^ during 10–500 ns and 200.50 ± 2.84 nm^2^ during 400–500 ns, with a final value of 200.21 nm^2^. The highest SASA value within the Soluplus^®^ group was observed in the αM–Soluplus^®^ 1:7 system, with a mean value of 281.58 ± 3.42 nm^2^ during 10–500 ns and 281.67 ± 3.42 nm^2^ during 400–500 ns, with a final value of 285.57 nm^2^.

In the αM–Kollidon^®^ VA64 systems, the SASA values were distributed within a narrower range than those observed for Soluplus^®^. The αM–Kollidon^®^ VA64 1:1 system showed a mean SASA of 11.29 ± 1.59 nm^2^ during 10–500 ns and 11.27 ± 1.61 nm^2^ during 400–500 ns, with a final value of 10.91 nm^2^. The αM–Kollidon^®^ VA64 1:3 system showed a mean SASA of 11.56 ± 1.61 nm^2^ during 10–500 ns and 11.55 ± 1.64 nm^2^ during 400–500 ns, with a final value of 13.09 nm^2^. The αM–Kollidon^®^ VA64 1:5 system exhibited a mean SASA of 11.81 ± 1.64 nm^2^ during 10–500 ns and 11.78 ± 1.63 nm^2^ during 400–500 ns, with a final value of 10.39 nm^2^. The αM–Kollidon^®^ VA64 1:7 system showed the highest mean SASA within the Kollidon^®^ VA64 group, with values of 12.10 ± 1.64 nm^2^ and 12.14 ± 1.64 nm^2^ during 10–500 ns and 400–500 ns, respectively, and a final value of 14.98 nm^2^.

### 2.6. Time-Resolved Hydrogen Bond Profiles of αM–Polymer Systems

**Figure 7:**
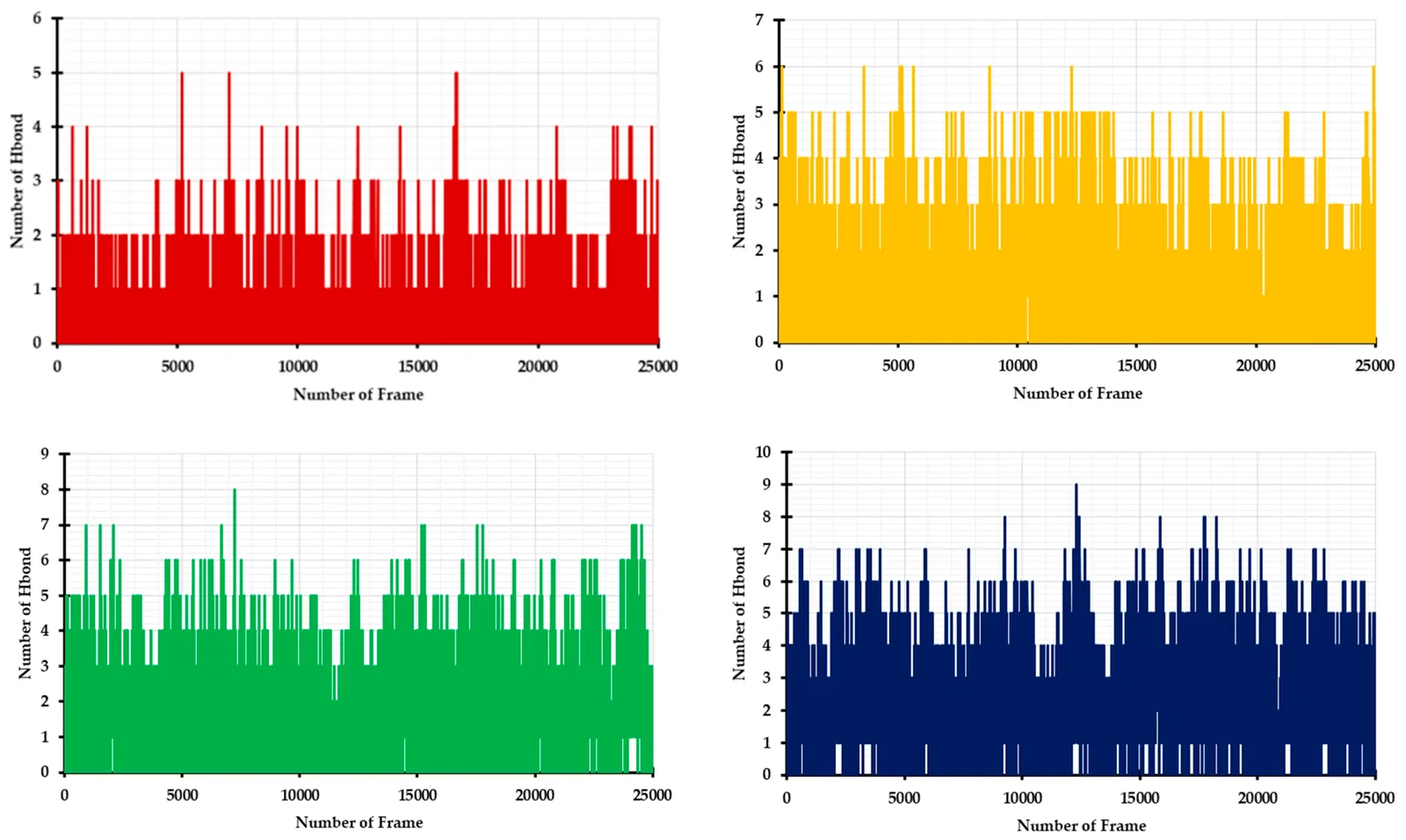
Time-resolved hydrogen bond distribution of the αM–Soluplus^®^ systems, showing the interaction frequency and stability across composition ratios of 1:1 (red), 1:3 (yellow), 1:5 (green), and 1:7 (blue).

The time-resolved hydrogen bond profiles of the αM–Soluplus^®^ systems are shown in Figure 7. The number of hydrogen bonds fluctuated throughout the simulation, with different maximum values observed for different composition ratios. In the αM–Soluplus^®^ 1:1 system, the hydrogen bond number was generally distributed between 0 and 3, with occasional increases up to five. The 1:3 system exhibited a broader distribution, with hydrogen bond numbers frequently observed between 1 and 4, reaching a maximum of 6. In the 1:5 system, the number of hydrogen bonds was generally higher, with values commonly distributed between 3 and 5 and occasional increases of up to 8. The 1:7 system showed the highest time-resolved hydrogen-bond profile within the Soluplus^®^ group, with frequent values between 4 and 6 and a maximum of 9 hydrogen bonds.

**Figure 8:**
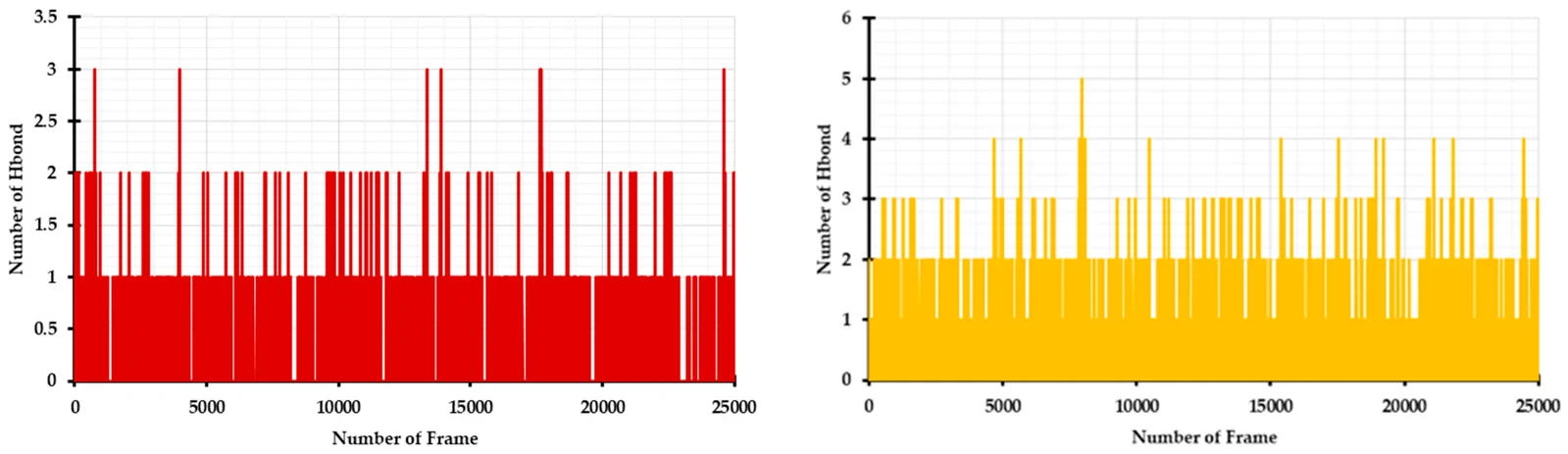

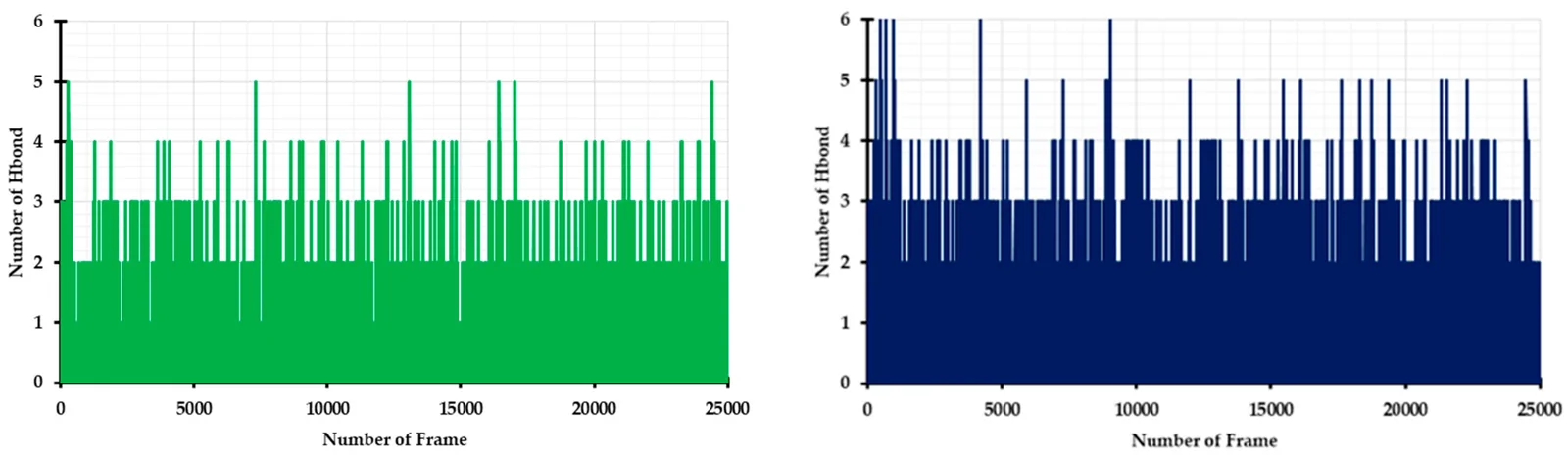
Time-resolved hydrogen bond profiles of the αM–Kollidon® VA64 systems, showing the interaction frequency and stability across composition ratios of 1:1 (red), 1:3 (yellow), 1:5 (green), and 1:7 (blue).

The time-resolved hydrogen-bond profiles of the αM–Kollidon^®^ VA64 systems are presented in Figure 8. Compared with the Soluplus^®^ systems, the Kollidon^®^ VA64 systems showed fewer hydrogen bond numbers across all simulation frames. In the αM–Kollidon^®^ VA64 1:1 system, the hydrogen bond numbers were mostly distributed between 0 and 2, with occasional increases up to 3. The 1:3 system showed values generally distributed between 1 and 3, with a maximum of five hydrogen bonds. The 1:5 system showed a similar upper range, with hydrogen bond numbers commonly observed between 1 and 3 and occasional increases of up to 5. The 1:7 system showed the highest hydrogen bond profile within the Kollidon^®^ VA64 group, with values frequently distributed between 2 and 4, reaching a maximum of 6 hydrogen bonds.

### 2.7. Quantitative Hydrogen Bond Occupancy in αM–Polymer Systems

The quantitative analysis of the hydrogen-bond pairs and cumulative H-bond occupancy in the αM–Soluplus^®^ systems is listed in Table 1. The number of hydrogen bond pairs increased with increasing Soluplus^®^ ratio. At a 1:1 ratio, 200 hydrogen-bond pairs were identified, with a cumulative occupancy of 52.54%. This number increased to 578 pairs at a 1:3 ratio with a cumulative occupancy of 130.22%, and further increased to 924 pairs at a 1:5 ratio with a cumulative occupancy of 179.09%. The highest value was observed at the 1:7 ratio, with 1150 hydrogen bond pairs and a cumulative occupancy of 231.17%.

The quantitative hydrogen bond results for the αM–Kollidon^®^ VA64 systems are listed in Table 2. The number of hydrogen-bond pairs also increased with increasing polymer ratio. At a 1:1 ratio, the system exhibited 128 hydrogen bond pairs, with a cumulative occupancy of 16.07%. This value increased to 411 pairs at the 1:3 ratio (cumulative occupancy of 46.19%) and to 653 pairs at the 1:5 ratio (cumulative occupancy of 71.26%). The 1:7 ratio showed the highest value within the Kollidon^®^ VA64 group, with 900 hydrogen bond pairs and a cumulative occupancy of 96.69%.

### 2.8. MM/PBSA-Based Binding Free Energy Analysis of αM–Polymer Systems

The binding energy profiles calculated using the MM/PBSA method for the αM–Soluplus^®^ and αM–Kollidon^®^ VA64 systems are presented in Table 3 and Table 4, respectively. The energetic components analyzed included van der Waals, electrostatic, polar solvation, SASA, and total binding energies. Overall, all systems showed negative total binding energies for the investigated composition ratios.

In the αM–Soluplus^®^ systems, the total binding energy became progressively more negative with increasing polymer ratio. The αM–Soluplus^®^ 1:1 system exhibited a binding energy of −126.24 ± 63.57 kJ/mol. At a 1:3 ratio, the binding energy decreased to −297.72 ± 90.06 kJ/mol. A more negative value was observed at the 1:5 ratio, with a binding energy of −430.09 ± 95.31 kJ/mol, while the 1:7 system showed the most negative binding energy within the Soluplus^®^ group, with a value of −484.09 ± 101.77 kJ/mol. A similar trend was observed for the van der Waals energy, which changed from −154.12 ± 74.63 kJ/mol at the 1:1 ratio to −571.56 ± 122.35 kJ/mol at the 1:7 ratio. The electrostatic energy also showed negative values across all ratios, ranging from −23.49 ± 20.42 to −105.50 ± 41.59 kJ/mol. In contrast, the polar solvation energy showed positive values and increased from 74.31 ± 41.21 kJ/mol at the 1:1 ratio to 277.12 ± 71.78 kJ/mol at the 1:7 ratio.

In the αM–Kollidon^®^ VA64 systems, the total binding energy became progressively more negative as the polymer ratio increased. The αM–Kollidon^®^ VA64 1:1 system exhibited a binding energy of −71.58 ± 40.28 kJ/mol. At the 1:3 ratio, the binding energy decreased to −210.83 ± 67.59 kJ/mol. The 1:5 system showed a binding energy of −302.84 ± 84.87 kJ/mol, whereas the 1:7 system exhibited the most negative binding energy within the Kollidon^®^ VA64 group, with a value of −382.41 ± 86.58 kJ/mol. The van der Waals energy became more negative, changing from −75.07 ± 41.61 kJ/mol at the 1:1 ratio to −427.05 ± 95.44 kJ/mol at the 1:7 ratio. The electrostatic energy remained negative across all ratios, ranging from −7.55 ± 10.90 to −49.08 ± 26.45 kJ/mol. In contrast, the polar solvation energy remained positive and increased from 24.42 ± 21.47 kJ/mol at the 1:1 ratio to 165.20 ± 48.73 kJ/mol at the 1:7 ratio.

## 3. Discussion

The physical stability of an ASD arises from the interplay between drug–polymer miscibility, molecular mobility, and the nature of the intermolecular interactions established within the polymer matrix. Hydrogen bonding, hydrophobic association, van der Waals contacts, and steric confinement may collectively restrict drug diffusion and reduce the probability of molecular rearrangement required for nucleation and crystal growth [42,43]. In the present study, MD simulations were employed to examine how αM was accommodated within Soluplus^®^ and Kollidon^®^ VA64 matrices across different drug-to-polymer ratios. The resulting trajectories provide comparative molecular-level information on drug–polymer association, structural organization, local mobility, compactness, solvent exposure, and interaction energetics. However, these findings should not be regarded as direct evidence of ASD formation or long-term physical stability, both of which require confirmation by experimental solid-state characterization. Rather, the simulations offer a mechanistic basis for assessing polymer suitability during the early stages of ASD development by identifying formulation-dependent differences in molecular organization and non-covalent interaction patterns [29,30,44]. This interpretation is consistent with recent computational studies showing that MD simulations can support the rationalization of ASD stabilization by relating drug loading and polymer composition to molecular mobility and drug–polymer interactions at the atomistic level [45].

### 3.1. Polymer- and Ratio-Dependent Structural Stabilization of αM

The RMSD profiles indicated that all αM–polymer systems reached relatively stable configurations during the 500 ns simulations, although the stabilization pattern depended on the polymer type and composition. RMSD is commonly used in ASD-related MD studies to monitor structural evolution and determine whether a simulated system maintains a consistent configuration over time [29,30]. Among the Soluplus^®^ systems, the 1:3 ratio showed the lowest mean RMSD, with values of 3.6968 ± 0.0255 nm over 10–500 ns and 3.6924 ± 0.0273 nm over 400–500 ns, together with a final value of 3.7037 nm. The 1:7 system also remained stable but exhibited slightly higher values of 3.9650 ± 0.0301 and 3.9679 ± 0.0277 nm over the corresponding intervals. These results suggest that increasing the Soluplus^®^ fraction beyond 1:3 did not further improve global structural stability, although a higher polymer content may still increase the availability of local drug–polymer contacts.

A different trend was observed for Kollidon^®^ VA64, for which the lowest RMSD occurred at the 1:7 ratio. This system showed mean values of 4.0120 ± 0.0274 nm over 10–500 ns and 4.0117 ± 0.0268 nm over 400–500 ns, with a final RMSD of 4.0314 nm. Thus, Kollidon^®^ VA64 appears to require a higher polymer fraction to achieve its most stable global configuration. The contrasting behavior of the two polymers may reflect differences in molecular architecture: Soluplus^®^ is an amphiphilic graft copolymer containing polyvinyl caprolactam, polyvinyl acetate, and polyethylene glycol segments, whereas Kollidon^®^ VA64 is a vinylpyrrolidone–vinyl acetate copolymer whose stabilizing performance is strongly influenced by drug–polymer compatibility [46,47]. Overall, the RMSD results indicate that Soluplus^®^ provided favorable global stabilization at a lower polymer ratio, whereas Kollidon^®^ VA64 benefited from increased polymer loading.

The RMSF results showed that global stability and local flexibility were not directly correlated. In the Soluplus^®^ systems, the mean RMSF increased from 2.5264 ± 0.0297 nm at 1:1 to 2.8185 ± 0.0601 nm at 1:7, while the 1:3 system showed an intermediate value of 2.6253 ± 0.0418 nm. The higher fluctuation at 1:7 may be associated with the greater contribution of flexible Soluplus^®^ segments and broader molecular distribution. Accordingly, the 1:3 ratio exhibited the most balanced profile, combining the lowest RMSD with a moderate local mobility. This finding is consistent with the view that ASD stabilization depends not only on the extent of drug–polymer contact, but also on the capacity of the polymer environment to restrict excessive molecular motion [29,44].

A comparable increase in RMSF was observed for Kollidon^®^ VA64, from 2.5078 ± 0.0396 nm at 1:1 to 2.7195 ± 0.0413 nm at 1:7. Although the 1:7 system showed the lowest RMSD within the Kollidon^®^ series, it also exhibited the highest local fluctuation. Therefore, it should be interpreted as having the most stable global configuration but greater segmental mobility, rather than as the least dynamic system. Compared with Soluplus^®^ 1:7, its lower RMSF suggests a relatively more rigid local environment, which may be related to differences in polymer architecture and segmental flexibility [46,47].

### 3.2. Molecular Compactness, Spatial Distribution, and Surface Exposure

The RDF analysis was used to assess the preferred intermolecular distances between αM and the surrounding polymer components during the simulations. A pronounced peak at a short distance generally reflects a localized drug–polymer association, whereas a lower peak or a shift toward a longer distance may indicate that the contacts are distributed more broadly throughout the polymer matrix. This distinction is relevant to ASD models because molecular stabilization depends not only on the occurrence of drug–polymer interactions, but also on how the drug is spatially accommodated within the carrier environment [29,30]. In the Soluplus^®^ systems, the RDF profiles changed progressively with increasing polymer content. The 1:1 system showed the highest maximum g(r), reaching 1.315 at 0.834 nm, whereas the peak decreased to 1.029 and shifted to 0.890 nm at the 1:7 ratio. This pattern suggests a transition from relatively localized contacts at lower polymer content to a broader distribution of αM within the Soluplus^®^ matrix at higher ratios. Accordingly, the lower RDF peak observed for Soluplus^®^ 1:7 should not be interpreted solely as a weaker association, but rather as evidence of a more dispersed interaction environment, consistent with the higher Rg and RMSF values recorded for this system. Similar molecular simulation studies have shown that favorable API–polymer interactions may promote miscibility and kinetic stabilization by reducing drug reassociation and recrystallization [48]. This behavior is also consistent with the amphiphilic graft architecture of Soluplus^®^, comprising polyvinyl caprolactam, polyvinyl acetate, and polyethylene glycol segments, which has been widely employed to improve molecular dispersion and suppress recrystallization in ASD systems [46,49,50,51].

The Kollidon^®^ VA64 systems displayed a more confined RDF pattern, with peak positions distributed within a narrow range of 0.812–0.830 nm. The maximum g(r) values were similar at the 1:1 and 1:3 ratios, at 1.136 and 1.137, respectively, and decreased to 1.103 at 1:5 and 1.067 at 1:7. Compared to Soluplus^®^, this narrower distribution suggests that αM remained within a more consistent interaction distance in the Kollidon^®^ VA64 environment. This finding is consistent with the Rg results, which indicate a comparatively more compact molecular organization for Kollidon^®^ VA64. This behavior may be associated with the vinylpyrrolidone–vinyl acetate structure of the polymer, in which lactam and acetate carbonyl groups can participate in drug–polymer interactions and contribute to amorphous stabilization. Previous studies have similarly shown that Kollidon^®^ VA64 can inhibit recrystallization and improve ASD performance, although its effectiveness remains strongly dependent on drug–polymer compatibility and composition [46,52,53]

The radius of gyration (Rg) was evaluated to describe changes in the overall spatial organization of the αM–polymer assemblies. A lower Rg generally indicates a more compact mass distribution, whereas a higher value reflects a more expanded arrangement. However, in polymer-containing systems, increases in Rg must be interpreted cautiously because they may arise not only from reduced compactness, but also from higher polymer content, greater segmental flexibility, and a broader spatial distribution of drug molecules [29,30]. In the Soluplus^®^ systems, the mean Rg increased from 2.5281 ± 0.0165 nm at 1:1 to 2.8334 ± 0.0234 nm at 1:7 during 10–500 ns. The same trend was observed over 400–500 ns, with values of 2.5287 ± 0.0145 and 2.8311 ± 0.0204 nm, respectively. These results indicate that increasing the Soluplus^®^ fraction produced a broader and more open molecular arrangement rather than a more compact assembly. This interpretation is consistent with the RMSF data, in which the higher Soluplus^®^ ratios exhibited greater local mobility, likely because of the increased contribution of flexible polymer segments and the wider distribution of αM within the matrix.

The Kollidon^®^ VA64 systems followed the same general trend, although the increase in Rg was less pronounced. The mean value increased from 2.5116 ± 0.0133 nm at 1:1 to 2.7226 ± 0.0101 nm at 1:7, with comparable values maintained during the final 400–500 ns. Relative to Soluplus^®^, the narrower Rg range and smaller deviations suggest that Kollidon^®^ VA64 formed a more compact molecular organization. Thus, the two polymers appeared to accommodate αM differently: Soluplus^®^ promoted a broader and more dynamic arrangement, consistent with its amphiphilic graft structure, whereas Kollidon^®^ VA64 maintained a comparatively tighter molecular environment.

Solvent-accessible surface area (SASA) analysis was used to evaluate the extent to which the αM–polymer assemblies were exposed to the surrounding simulation environment. In polymeric systems, a higher SASA may reflect a more open molecular arrangement and a greater contribution from exposed polymer segments; however, it should not be interpreted independently as an indicator of improved stability. Therefore, SASA is most informative when considered together with the RMSD, RMSF, Rg, RDF, hydrogen bonding, and MM/PBSA results [29,30]. In the Soluplus^®^ systems, the mean SASA increased markedly from 48.42 ± 1.90 nm^2^ at 1:1 to 281.58 ± 3.42 nm^2^ at 1:7 during 10–500 ns. A similar pattern was observed over 400–500 ns, with values increasing from 48.46 ± 1.93 to 281.67 ± 3.42 nm^2^. This pronounced increase indicates that higher Soluplus^®^ content produced a more exposed and spatially expanded molecular arrangement, consistent with the corresponding Rg and RMSF profiles. Therefore, the 1:7 system may provide a larger molecular contact environment, although this does not necessarily imply greater global structural stability than the 1:3 system.

In contrast, the Kollidon^®^ VA64 systems showed substantially lower and less variable SASA values. The mean SASA increased only slightly from 11.29 ± 1.59 nm^2^ at 1:1 to 12.10 ± 1.64 nm^2^ at 1:7 during 10–500 ns, while the corresponding values over 400–500 ns ranged from 11.27 ± 1.61 to 12.14 ± 1.64 nm^2^. This narrower range suggests that Kollidon^®^ VA64 maintained a less expansive molecular environment than Soluplus^®^. The difference was most evident at the 1:7 ratio, where Soluplus^®^ showed a mean SASA of 281.58 ± 3.42 nm^2^, whereas Kollidon^®^ VA64 remained at 12.10 ± 1.64 nm^2^. Overall, these results indicate that Soluplus^®^ formed a more open and exposed molecular arrangement, whereas Kollidon^®^ VA64 preserved a comparatively denser organization. This distinction reflects differences in spatial distribution and molecular exposure; therefore, the relative strength of stabilization must be interpreted together with the hydrogen bonding and MM/PBSA analyses.

### 3.3. Drug–Polymer Interaction Network and Binding Energetics

Hydrogen-bond analysis revealed that increasing the polymer fraction enhanced both the frequency and extent of αM–polymer interactions, with a more pronounced effect observed in the Soluplus^®^ systems. In ASD models, hydrogen bonding may restrict drug mobility and reduce the likelihood of self-association. However, stabilization cannot be attributed to hydrogen bonding alone, because hydrophobic contacts, van der Waals interactions, and steric confinement within the polymer environment may also contribute substantially [29,44]. In the αM–Soluplus^®^ systems, the 1:1 ratio generally formed 0–3 hydrogen bonds per frame, with occasional values up to 5, whereas the 1:7 system frequently formed 4–6 hydrogen bonds and reached a maximum of 9. The quantitative analysis showed the same trend, with the number of hydrogen-bond pairs increasing from 200 at 1:1 to 1150 at 1:7 and the cumulative occupancy rising from 52.54% to 231.17%. These findings suggest that a higher Soluplus^®^ content provides a larger number of accessible interaction sites for αM, particularly through carbonyl, ether, and ester acceptor groups. Nevertheless, the extensive hydrogen-bond network in the 1:7 system should not be interpreted as evidence of superior stability across all descriptors because the lowest RMSD was observed at the 1:3 ratio.

The Kollidon^®^ VA64 systems exhibited a similar polymer-dependent increase in hydrogen bonding, although the magnitude remained lower than that observed for Soluplus^®^. In the 1:1 system, hydrogen bonds were generally distributed between 0 and 2 per frame, with a maximum of 3, whereas the 1:7 system frequently showed 2–4 hydrogen bonds and reached a maximum of 6. Across the same composition range, the number of hydrogen-bond pairs increased from 128 to 900, whereas the cumulative occupancy increased from 16.07% to 96.69%. These results indicate that Kollidon^®^ VA64 can establish hydrogen-bond interactions with αM, primarily through the carbonyl groups of its vinylpyrrolidone and vinyl acetate units, although its overall interaction capacity is lower than that of Soluplus^®^. Among all systems, Soluplus^®^ 1:7 exhibited the most extensive and persistent hydrogen-bond network. This behavior is consistent with the amphiphilic graft architecture of Soluplus^®^, which provides both polar and nonpolar domains capable of supporting hydrogen bonding, hydrophobic association, and dispersion interactions with the xanthone scaffold of αM. Therefore, the hydrogen bond results support a stronger interaction network in the Soluplus^®^ systems, although their interpretation should be integrated with MM/PBSA as an energetic descriptor of drug–polymer association [46,47].

The MM/PBSA analysis provided an energetic perspective complementary to the structural, RDF, and hydrogen-bond results. All systems exhibited negative interaction-energy estimates, indicating energetically favorable αM–polymer association within the simulated models. However, these values should be interpreted comparatively rather than as absolute thermodynamic binding free energies because they are influenced by continuum-solvation assumptions, force-field dependence, and the omission of entropic contributions [32,54].

For the αM–Soluplus^®^ systems, the estimated interaction energy became progressively more negative as the polymer ratio increased, changing from −126.24 ± 63.57 kJ/mol at 1:1 to −484.09 ± 101.77 kJ/mol at 1:7. Van der Waals interactions provided the dominant favorable contribution, decreasing from −154.12 ± 74.63 to −571.56 ± 122.35 kJ/mol over the same ratio range. This trend is consistent with the hydrophobic xanthone framework of αM, for which nonpolar contacts and dispersion forces are expected to contribute substantially to the polymer association. Electrostatic interactions were also favorable at all ratios, ranging from −23.49 ± 20.42 to −105.50 ± 41.59 kJ/mol, indicating additional polar contributions, including those associated with hydrogen bonding. In contrast, the polar solvation term was unfavorable and increased to 277.12 ± 71.78 kJ/mol at 1:7, but this penalty was outweighed by the more favorable van der Waals and SASA contributions.

A comparable trend was observed in the αM–Kollidon^®^ VA64 systems, although the overall energetic association was less favorable than that of Soluplus^®^. The estimated interaction energy decreased from −71.58 ± 40.28 kJ/mol at 1:1 to −382.41 ± 86.58 kJ/mol at 1:7. Van der Waals energy was again the major favorable component, changing from −75.07 ± 41.61 to −427.05 ± 95.44 kJ/mol, whereas electrostatic energy ranged from −7.55 ± 10.90 to −49.08 ± 26.45 kJ/mol. The polar solvation contribution remained positive and increased from 24.42 ± 21.47 to 165.20 ± 48.73 kJ/mol. These findings confirm that Kollidon^®^ VA64 formed energetically favorable associations with αM, although its van der Waals, electrostatic, and total interaction energy contributions were consistently less favorable than those of Soluplus^®^ at corresponding ratios.

Among the investigated systems, Soluplus^®^ 1:7 exhibited the most negative non-entropic MM/PBSA interaction-energy estimate. The energetic profile was dominated by nonpolar contributions, demonstrating that αM–polymer association was not governed solely by hydrogen bonding. This interpretation agrees with the hydrogen-bond analysis, in which Soluplus^®^ 1:7 showed a greater number of hydrogen-bond pairs and higher cumulative occupancy than Kollidon^®^ VA64 1:7. Similar computational studies of amorphous formulations have shown that energetic analysis can complement structural descriptors by characterizing drug–excipient association patterns relevant to molecular organization in amorphous systems [55]. Importantly, however, the MM/PBSA ranking did not correspond directly to the RMSD results. Soluplus^®^ 1:7 showed the most favorable interaction-energy estimate and the most extensive hydrogen-bond network, whereas Soluplus^®^ 1:3 exhibited the lowest mean RMSD within the Soluplus^®^ series. This distinction indicates that local interaction strength and global trajectory stability are related but non-equivalent properties. Accordingly, Soluplus^®^ 1:3 was characterized by a comparatively stable global trajectory, whereas Soluplus^®^ 1:7 exhibited a denser local interaction network. These model-dependent findings do not establish ASD formation, formulation superiority, dissolution enhancement, or storage stability. Rather, they generate testable hypotheses that require confirmation through experimental solid-state characterization, dissolution and supersaturation studies, and physical-stability testing [56].

Overall, the simulations revealed polymer- and ratio-dependent differences in the molecular behavior of αM. Relative to the Kollidon^®^ VA64 models, the Soluplus^®^ systems generally exhibited more extensive hydrogen bonding interactions and more negative non-entropic MM/PBSA interaction energy estimates under the evaluated conditions. However, the lowest mean RMSD and the most favorable local interaction profile occurred at different Soluplus^®^ ratios, demonstrating that the global trajectory behavior and local interaction magnitude did not yield an identical ranking. Therefore, these findings should be interpreted as preliminary, model-dependent comparative observations rather than evidence supporting the selection of a particular polymer or formulation ratio.

## 4. Materials and Methods

In this study, αM, Soluplus^®^, and Kollidon^®^ VA64 were used as molecular components for the creation of polymer-based ASD models. To map the polymer- and ratio-dependent stabilization mechanisms, two different αM–polymer systems were developed: αM–Soluplus^®^ and αM–Kollidon^®^ VA64. The systems were constructed at drug:polymer ratios of 1:1, 1:3, 1:5, and 1:7. The molecular models were optimized for structure, system creation, in silico solvent evaporation, MD simulation, trajectory analysis, and MM/PBSA binding free energy calculations.

The chemical structure of αM was retrieved from the PubChem database, and Soluplus^®^ and Kollidon^®^ VA64 were represented by appropriate polymeric structural units. Two-dimensional structures were drawn using ChemDraw Professional 16.0 and transformed into three-dimensional geometries using Chem3D 16.0 software. Gaussian 16 was then used to optimize the molecular geometries. PACKMOL version 20.15.2 was used to build the initial molecular configurations, where regulated spatial distributions of αM, polymeric carriers, and methanol were obtained in the simulation box. Methanol was used as a temporary solvent to allow the molecules to disperse homogeneously before developing solvent-free ASD models. After equilibration in methanol, the solvent molecules were removed as an in silico approximation of solvent evaporation. The resulting solvent-free drug–polymer complexes were then subjected to energy minimization, equilibration, and production MD simulations using GROMACS 2016.3. The structural stability, molecular flexibility, compactness, solvent exposure, spatial distribution, hydrogen bonding, interaction energy, and binding free energy of the trajectories were analyzed. All calculations were performed on a workstation equipped with an Intel^®^ Core™ i5-8500 CPU @ 3.00 GHz (6 cores), 16 GB DDR4 2667 MHz RAM, 2 TB hard disk drive (HDD), 120 GB solid-state drive (SSD), and NVIDIA GeForce GTX 1080 Ti graphics card.

### 4.1. Preparation and Structural Optimization of Drug–Polymer Components

**Figure 9:**
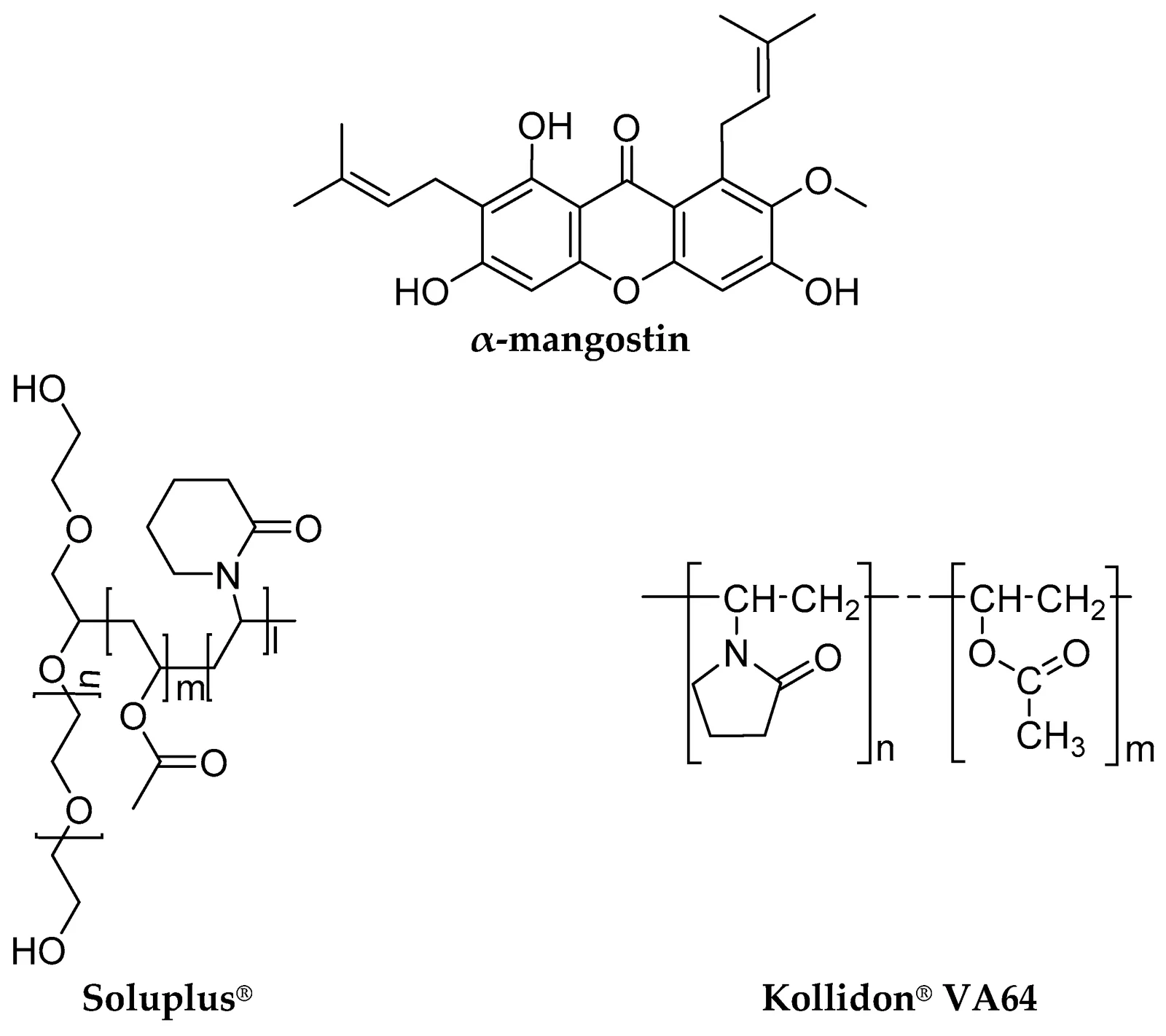
Two-dimensional visualization of αM, Soluplus^®^, and Kollidon^®^ VA64 for the development of drug–polymer amorphous solid dispersion systems.

Structurally accurate input geometries for the MD simulations were obtained through molecular preparation. αM (PubChem CID: 5281650), Soluplus^®^, and Kollidon^®^ VA64 were used as the molecules in the ASD systems (Figure 9). The structure of αM was obtained from the PubChem database (https://pubchem.ncbi.nlm.nih.gov; accessed on 18 October 2025) [57]. Soluplus^®^ was modeled as a representative amphiphilic graft copolymer consisting of polyvinyl caprolactam, polyvinyl acetate, and polyethylene glycol segments, whereas Kollidon^®^ VA64 was modeled as a representative copovidone structure comprising N-vinylpyrrolidone and vinyl acetate repeating units. Representative oligomeric models were employed to preserve the characteristic functional groups and polarity of the commercial polymers while maintaining computational feasibility for atomistic MD simulations. The importance of this differential is that both polymers have different functional groups and polarity profiles, which may affect their interaction with αM. αM was represented using its complete molecular structure, with the molecular formula C_24_H_26_O_6_ and a molecular weight of 410.46 g/mol. In contrast, the polymeric carriers were represented using one type of simplified composite computational structural unit for each polymer. The representative Soluplus^®^ structural unit contained one N-vinylcaprolactam unit, one vinyl acetate unit, and one ethylene oxide unit, giving a combined molecular formula of C_14_H_23_NO_4_ and a calculated molecular weight of 269.34 g/mol. The representative Kollidon^®^ VA64 structural unit contained one N-vinyl-2-pyrrolidone unit and one vinyl acetate unit, giving a combined molecular formula of C_10_H_15_NO_3_ and a calculated molecular weight of 197.23 g/mol. These molecular weights refer exclusively to the simplified computational structural units used in the present model and should not be interpreted as the average molecular weights or complete chain architectures of commercial Soluplus^®^ and Kollidon^®^ VA64.

The two-dimensional structures of αM and the polymeric units were drawn and checked for the proper depiction of functional groups and bonding patterns using ChemDraw Professional v.16.0. The structures were subsequently translated into 3D conformations using Chem3D 16.0. Prior to quantum mechanical optimization, preliminary energy minimization was performed to reduce unfavorable local geometries. These compounds were optimized using the Gaussian 16 software package. The molecular geometries were refined in the gas phase using density functional theory (DFT). Specifically, the B3LYP functional combined with the 6-31G(d) basis set was applied for all quantum chemical calculations [58], ensuring accurate and reliable structural optimization of molecules.

After geometry optimization, hydrogen atoms were added, atomic charges were allocated, and the topology was prepared. The first structure preparation was performed using AutoDock Tools 4.2.6 in the MGLTools 1.5.6 environment [59]. Parameters consistent with the topology and force field were created using ACPYPE and the AMBER99SB-ILDN force field for MD simulations. ACPYPE was used to generate GROMACS-compatible molecular topologies, including bonded and non-bonded interaction parameters and partial atomic charges. The generated topologies were carefully inspected to ensure chemically reasonable molecular connectivity and geometrical parameters before MD simulations. Although AMBER99SB-ILDN was originally developed for biomolecular systems, it was employed to maintain compatibility between the ACPYPE-generated topologies and the GROMACS simulation framework. Therefore, the present simulations were intended to comparatively evaluate the molecular interactions between αM and the polymeric carriers rather than to reproduce the bulk physicochemical properties of the polymers. This phase was necessary to establish consistency between the molecular geometry, partial charges, bonded parameters, and nonbonded interaction terms used in the subsequent GROMACS simulations.

### 4.2. Optimization of Initial System Configuration for αM–Polymer Systems

The initial ASD systems were constructed using an in silico solvent-evaporation approach. Methanol was selected as the provisional solvent because of its ability to disperse hydrophobic αM molecules and polymeric components, thereby facilitating molecular-level mixing before solvent removal. Eight αM molecules were included in each simulation system and maintained constant across all formulations to enable consistent comparisons between polymer types and drug-to-polymer ratios. The polymer content was subsequently adjusted to represent target drug-to-polymer mass ratios of 1:1, 1:3, 1:5, and 1:7, as summarized in Table 5 and Table 6. This systematic increase in the polymer fraction enabled the evaluation of its influence on molecular organization, system compactness, and drug–polymer interactions within the ASD models.

The number of simplified polymer structural units required for each system was calculated using the following equation:

$$N_{polymer}=N_{\alpha M}\times \frac{M_{\alpha M}}{M_{polymer\;unit}}\times R$$

where $N_{\alpha M}$ is the number of αM molecules, $M_{\alpha M}$ is the molecular weight of αM, $M_{polymer\;unit}$ is the molecular weight of the simplified computational polymer unit, and R is the target polymer-to-drug mass ratio. The calculated non-integer values were rounded to whole structural-unit counts for system construction. The resulting actual mass ratios and detailed molecular composition calculations are provided in Table S1.

The initial molecular configurations were produced using PACKMOL version 20.15.2 [60]. The random placement of αM, polymer units, and methanol molecules was carried out in a cubic simulation box with a minimum tolerance distance of 2 Å between atoms to avoid excessive steric overlap. The molecular components were placed in a cubic simulation box with dimensions of 10 × 10 × 10 nm^3^. Periodic boundary conditions were applied in all three spatial dimensions to simulate a bulk-like system.

The solvent evaporation protocol was implemented by first constructing methanol-containing systems composed of αM, polymer units, and methanol. The solvated systems were subjected to energy minimization and equilibration to allow molecular redistribution in the solvent environment. After equilibration, the methanol molecules were removed from the system to generate solvent-free αM–polymer matrices. This step was used as an in silico approximation of the solvent evaporation during the ASD formation. The solvent-free systems were then subjected to a second energy minimization and equilibration step to allow structural relaxation before the production of MD simulations. This workflow was designed to promote close molecular contact between αM and the polymeric carrier while minimizing artifacts arising from unfavorable initial molecular placements.

### 4.3. Molecular Dynamics Simulation of αM–Polymer Systems

MD simulations were performed to study the structural stability and intermolecular interactions of αM in Soluplus^®^ and Kollidon^®^ VA64 matrices in the context of solvent-free ASD. All simulations were performed using GROMACS 2016.3 [61,62]. The initial configurations were considered as the optimized and equilibrated structures produced during the system preparation process. The steepest descent technique was originally applied for energy minimization to reduce unfavorable connections and steric conflicts. Minimization was performed over 500 steps with a convergence threshold of 10 kJ mol^−1^ nm^−1^ and a maximum step size of 0.01 nm. Periodic boundary conditions were used in all three spatial directions [63]. A grid-based approach was used for the neighbor search. For minimization, a cutoff distance of 1.5 nm was adopted for both the Coulombic and van der Waals interactions. Long-range electrostatic interactions were handled using the PME approach with a grid spacing of 0.1 nm [64]. Van der Waals interactions were treated using a switching function from 1.1 to 1.5 nm, and a dispersion correction was performed to account for long-range energy and pressure contributions. All covalent bonds involving hydrogen atoms were constrained using the LINCS algorithm, allowing the use of a 2 fs integration time step throughout the equilibration and production simulations [65].

Following minimization, each system was sequentially equilibrated in the constant volume and temperature (NVT) and constant pressure and temperature (NPT) ensembles. The NVT equilibration was run for 250,000 steps with a time step of 2 fs, which is 0.5 ns. The temperature was maintained at 298 K using a Berendsen thermostat with a coupling constant of 1 ps [66]. Subsequently, NPT equilibration was performed for another 250,000 steps with the same time step. The pressure was kept constant at 1 bar by employing the Berendsen barostat with a coupling constant of 0.5 ps and a compressibility of 5 × 10^−5^ bar^−1^. The Berendsen coupling algorithms were employed only during the equilibration stages to facilitate rapid stabilization of the system prior to production simulations. MD simulations were run during a production phase of 250,000,000 steps with a time step of 2 fs, which corresponds to a total simulation time of 500 ns. Temperature was maintained at 298 K during production using the velocity-rescaling thermostat, whereas isotropic pressure coupling was performed using the Parrinello–Rahman barostat with a coupling constant of 2.0 ps and a reference pressure of 1 bar to ensure proper sampling of the NPT ensemble. Short-range electrostatic and van der Waals interactions were estimated with a cutoff distance of 1.3 nm. The neighbor list was updated every 10 steps using the grid-based technique. Trajectory coordinates were recorded every 10,000 steps for further analysis. All bond lengths were restricted in the production simulation using the LINCS technique. This combination of thermostat and barostat was selected to provide rigorous temperature and pressure control during the production phase, thereby improving the reliability of the structural and dynamic properties obtained from the simulations.

### 4.4. Post-Simulation Analysis of Molecular Dynamics

Post-simulation analyses were performed to analyze the molecular behavior of the αM–polymer complexes along the production trajectories. The analyses obtained parameters such as root mean square deviation (RMSD), root mean square fluctuation (RMSF), radial distribution function (RDF), radius of gyration (Rg), solvent-accessible surface area (SASA), hydrogen bonding, and interaction energy. These parameters were chosen to facilitate the mechanistic interpretation introduced in the Introduction section, where the stabilization of ASD was assumed to depend on the structural stability, molecular flexibility, compactness, solvent exposure, spatial proximity, specific drug–polymer interactions, and energetic contributions.

The trajectories were examined using the integrated tools of GROMACS 2016.3. Before the analysis, periodic boundary artifacts were rectified, and the trajectories were adjusted using least squares to eliminate translational and rotational motions. The production trajectories were processed and analyzed after the removal of periodic boundary artifacts. The RMSD was used to assess global structural stability over time [67], whereas the RMSF was used to evaluate local atomic or segmental flexibility [68]. Rg was used to determine the compactness of the drug–polymer system [69]. SASA was employed to evaluate the exposure of αM to its surrounding environment [70]. Radial distribution function (RDF) analysis was performed to ascertain the preferential spatial distribution between the chosen atoms or functional groups of αM and the polymer matrix [71]. An investigation of hydrogen bonds was conducted to ascertain the quantity and stability of specific intermolecular interactions between αM and the selected polymers [72]. The interaction energy was computed to evaluate the significance of non-covalent interactions between αM and the polymeric carrier.

#### 4.4.1. Root Mean Square Deviation (RMSD)

The RMSD was calculated to evaluate the global structural stability of each αM–polymer system over the simulation time. The trajectory was fitted to a reference structure prior to the RMSD calculation to remove global translational and rotational motions. A lower and more stable RMSD profile was interpreted as an indication of improved dynamic stability of the drug–polymer complex. The RMSD was calculated using the following equation:

$$RMSD=\sqrt{\frac{\sum_{i=1}^{N}m_{i}(r_{i}(t)-r_{i}(t_{0}){)}^{2}}{\sum_{i=1}^{N}m_{i}}}$$

where N is the number of atoms, *m_i_* is the mass of atom *i*, *r_i_*(*t*) is the position at time *t*, and *r_i_*(*t*_0_) is the reference position.

#### 4.4.2. Root Mean Square Fluctuation (RMSF)

The RMSF was used to evaluate the flexibility of individual atoms or molecules over the simulation time. It provides information on the local dynamic behavior within the system. Higher RMSF values indicate greater atomic mobility. This parameter is particularly useful for identifying flexible regions in the polymer chains. The RMSF is calculated based on time-averaged atomic positions and is expressed as:

$$RMSF_{i}=\sqrt{\frac{1}{T}\sum_{t=1}^{T}{\left(r_{i}(t)-\langle r_{i}\rangle \right)}^{2}}$$

where ⟨*r_i_*⟩ represents the average position of atom *i* over the simulation time.

#### 4.4.3. Radial Distribution Function (RDF)

The RDF was used to evaluate the spatial distribution and preferential interaction distance between αM and the functional groups of the polymer. The RDF provides information on the likelihood of locating specified atoms or groups at a distance (r) from the reference atom or group. In this study, RDF analysis was performed on the chosen donor or acceptor atoms of αM and the groups of the polymer implicated in probable noncovalent interactions. This atom-pair-based approach is consistent with previous MD studies of drug–polymer or drug–material systems, in which the RDF was calculated using selected atoms and molecular centers of mass to evaluate the molecular affinity, preferential interaction distances, and orientation of small molecules within a polymeric environment [35]. The RDF profile peaks correspond to the preferred interaction distances and higher local molecular densities. The RDF was estimated using the following equation:

$$g(r)=\frac{1}{4\pi r^{2}\rho }\frac{dN}{dr}$$

where *ρ* is the number density, and *dN* represents the number of particles within a spherical shell at a distance *r*.

#### 4.4.4. Radius of Gyration (Rg)

The compactness of the molecular system was evaluated by calculating Rg. This represents the dispersion of atoms around the center of mass. A lower Rg value indicates a more compact molecular arrangement, whereas an increase in Rg indicates a more extended structure. For ASD systems, Rg provides information on the ability of the polymer matrix to sustain a compact drug–polymer assembly. Rg was determined using the following equation:

$$R_{g}=\sqrt{\frac{\sum_{i=1}^{N}m_{i}r_{i}^{2}}{\sum_{i=1}^{N}m_{i}}}$$

#### 4.4.5. Solvent-Accessible Surface Area (SASA)

SASA was calculated to evaluate the surface exposure of αM in the polymer matrix. This metric reflects the degree of exposure of the molecular surface to the surrounding environment. A lower SASA value indicates better shielding or encapsulation of αM by the polymer. A higher SASA value indicates greater exposure of the molecules. The SASA was determined as the total accessible surface area of all atoms:

$$SASA=\sum_{i=1}^{N}A_{i}$$

where *Ai* represents the solvent-accessible surface area of atom *i*. In this study, SASA was used to complement the interpretation of molecular compactness and drug exposure in each αM–polymer system.

#### 4.4.6. Hydrogen Bond Analysis

Hydrogen bond analysis was performed to evaluate the occurrence and persistence of specific interactions between αM and polymer matrices. Hydrogen bonds were identified using geometric criteria based on the donor–acceptor distance and donor–hydrogen–acceptor angle. A hydrogen bond was considered to be formed when the donor–acceptor distance was less than or equal to 0.30 nm and the donor–hydrogen–acceptor angle was greater than or equal to 150°. These criteria were consistently applied to all systems. The hydrogen-bond criterion can be expressed as follows:

$$H-bond=\left\{\begin{array}{c} 1,\;r_{DA}\leq 0.30\;nm\;and\;\theta \geq 150{}^{\circ}\\ 0,\;otherwise \end{array}\right.$$

where *r_DA_* represents the distance between the donor and acceptor atoms, and *θ* denotes the donor–hydrogen–acceptor angle. The number of hydrogen bonds was monitored throughout the production trajectory to evaluate the interaction stability and persistence. Persistent hydrogen bonding was interpreted as a potential molecular mechanism contributing to the stabilization of αM within the polymeric matrix.

### 4.5. MM/PBSA Binding Free Energy Analysis

MM/PBSA approach was used to estimate the binding free energy between αM and the polymer matrices based on molecular dynamics trajectories. This method combines molecular mechanics energy terms with solvation contributions and is commonly used to analyze the relative strengths of intermolecular interactions in molecular simulation studies [73]. In this study, MM/PBSA analysis was applied to compare the energetic contributions of Soluplus^®^ and Kollidon^®^ VA64 in stabilizing αM at different drug:polymer ratios. Representative snapshots from the equilibrated production trajectories were used for the binding free energy calculations. Before the calculation, the trajectory files were processed to remove periodic boundary artifacts. The same snapshot extraction protocol was applied to all systems to ensure comparability across polymer types and drug:polymer ratios. MM/PBSA calculations were performed using the g_mmpbsa tool integrated with the GROMACS workflow [74]. The total binding free energy was decomposed into several energetic components, including van der Waals interactions, electrostatic interactions, polar solvation energy, and nonpolar solvation energy. These components were analyzed to identify the dominant energetic contributions to αM–polymer stabilization.

The binding free energy (ΔGbinding) was calculated as the difference between the free energy of the αM–polymer complex and the sum of the free energies of the individual components of that complex. This relationship is expressed as ΔGbinding = Gcomplex − (Gdrug + Gpolymer), where Gcomplex, Gdrug, and Gpolymer represent the free energies of the αM–polymer complex, αM, and polymer matrix, respectively. The total free energy of each component was further decomposed into molecular mechanics and solvation energy terms, expressed as G = E_MM + G_solvation − TS, where E_MM represents the molecular mechanics energy, G_solvation comprises polar and nonpolar solvation energies, and TS represents the entropic contribution. In this study, the entropy term was not included because of its high computational cost and because the analysis was intended for a comparative interpretation among systems. This approximation is commonly adopted in comparative MM/PBSA studies, where relative binding free energies are of primary interest. However, the omission of the entropic contribution may influence the absolute binding free energy values, and in some cases, the relative ranking of binding affinities if the entropy contributions differ substantially among the investigated systems [75]. Therefore, the calculated ΔG_binding values should be interpreted primarily as comparative estimates of the enthalpic contributions to αM–polymer interactions, rather than absolute thermodynamic binding free energies [76]. The molecular mechanics energy term is further defined as E_MM = E_vdW + E_electrostatic, and the solvation energy is expressed as G_solvation = G_polar + G_nonpolar. The polar solvation energy was calculated using the Poisson–Boltzmann equation, and the nonpolar contribution was estimated based on the solvent-accessible surface area. More negative ΔGbinding values indicate more favorable αM–polymer interactions, whereas less negative or positive values indicate weaker or less favorable binding. The decomposition of the energy components was used to identify the dominant energetic contributions involved in αM stabilization within the polymeric ASD systems.

## 5. Conclusions

This study provides molecular-level insights into the interaction behavior of αM with Soluplus^®^ and Kollidon^®^ VA64 using molecular dynamics simulations and MM/PBSA analyses. Across the 500 ns simulations, all systems maintained relatively stable configurations, although the stabilization pattern varied with the polymer type and composition ratio. Soluplus^®^ 1:3 showed the most favorable global structural stability based on the RMSD value, whereas Kollidon^®^ VA64 1:7 showed the most stable system within the Kollidon^®^ series. Increasing the polymer ratio was generally associated with greater local flexibility, broader spatial distribution, and higher surface exposure, particularly in Soluplus^®^ systems. Interaction analyses further revealed that Soluplus^®^ provided a more favorable molecular environment for αM than Kollidon^®^ VA64. Soluplus^®^ 1:7 displayed the highest hydrogen-bonding capacity, with 1150 hydrogen-bond pairs and 231.17% cumulative occupancy, together with the most favorable MM/PBSA binding energy of −484.09 ± 101.77 kJ/mol. In comparison, Kollidon^®^ VA64 1:7 was the strongest system in its polymer group, with 900 hydrogen-bond pairs, 96.69% cumulative occupancy, and a binding energy of −382.41 ± 86.58 kJ/mol. These results indicate that Soluplus^®^ interacts more strongly with αM through a combination of van der Waals interactions, hydrogen bonding, and non-polar contacts.

Overall, the present findings represent a preliminary computational assessment of the relative interactions between αM and the investigated polymer systems, rather than definitive evidence of the amorphous solid dispersion performance. The observed molecular interaction patterns suggest that Soluplus^®^ 1:3 and 1:7 may serve as suitable candidates for subsequent experimental investigation, with the former exhibiting favorable global structural stability and the latter showing the strongest drug–polymer interaction profile under the simulated conditions. However, as this study was based entirely on computational simulations, further experimental validation through solid-state characterization, solubility and dissolution testing, and physical stability evaluation is essential to determine whether the predicted molecular interactions can be translated into experimentally achievable and pharmaceutically relevant ASD performance.

### Study Limitation Insights

This study had several limitations that should be considered when interpreting the results. First, αM–polymer systems were investigated using simplified computational conditions. Although the 500 ns simulations provide molecular-level information on the structural stability, flexibility, spatial organization, hydrogen bonding, and binding energy, the models cannot fully reproduce the complexity of experimentally prepared ASD systems. Furthermore, the simulation systems comprised a limited number of αM molecules and polymer repeat units, representing only a small-scale molecular model rather than the bulk amorphous solid dispersion encountered in pharmaceutical formulations. Consequently, the observed interactions primarily reflect local molecular behavior and should not be interpreted as a complete representation of the macroscopic properties of bulk ASD system. Factors such as solvent evaporation kinetics, polymer polydispersity, chain entanglement, residual solvent, humidity, mechanical processing, and long-term physical aging were not explicitly included in this study.

Second, the simulations were performed using classical force-field parameters that describe intermolecular interactions through predefined bonded and nonbonded terms. Consequently, the electronic polarization, charge transfer, and quantum mechanical contributions to hydrogen bonding were not fully represented. Similarly, the MM/PBSA binding energy should be interpreted as a comparative energetic estimate rather than an absolute thermodynamic value because the method relies on continuum solvation assumptions and a simplified treatment of solvent effects.

Finally, the molecular stability observed in the simulations should not be directly equated with experimental ASD formation, dissolution enhancement, or long-term storage stability. Instead, the present work should be regarded as a preliminary computational screening study that provides molecular-level insights into the relative interactions between αM and the investigated polymer systems. The observed differences among the formulations identify systems that warrant subsequent experimental investigation rather than establishing their pharmaceutical performance. Accordingly, experimental validation, including solid-state characterization, dissolution studies, and physical stability evaluation, remains essential to determine whether the predicted molecular interactions translate into an experimentally achievable ASD performance.

## Figures and Tables

**Table 1:** Quantitative analysis of hydrogen bond number and occupancy in αM–Soluplus^®^ systems across varying composition ratios.

Ratio (α-Mangostin:Polymer)	Number of H-Bonds	Cumulative H-Bond Occupancy (%)
1:1	200	52.54%
1:3	578	130.22%
1:5	924	179.09%
1:7	1150	231.17%

**Table 2:** Quantitative analysis of hydrogen bond number and occupancy in αM–Kollidon^®^ systems across varying composition ratios.

Ratio (α-Mangostin:Polymer)	Number of H-Bonds	Cumulative H-Bond Occupancy (%)
1:1	128	16.07%
1:3	411	46.19%
1:5	653	71.26%
1:7	900	96.69%

**Table 3:** Molecular interaction energy profile of αM–Soluplus^®^ systems from MM/PBSA calculations.

Ratio (αM:Polymer)	Van der Waals Energy (kJ/mol)	Electrostatic Energy (kJ/mol)	Polar Solvation Energy (kJ/mol)	SASA Energy (kJ/mol)	Binding Energy (kJ/mol)
1:1	−154.12 ± 74.63	−23.49 ± 20.42	74.31 ± 41.21	−22.94 ± 9.70	−126.24 ± 63.57
1:3	−355.07 ± 107.90	−59.52 ± 32.09	169.73 ± 66.24	−52.86 ± 13.73	−297.72 ± 90.06
1:5	−509.97 ± 115.84	−87.09 ± 36.62	241.81 ± 68.43	−74.85 ± 14.57	−430.09 ± 95.31
1:7	−571.56 ± 122.35	−105.50 ± 41.59	277.12 ± 71.78	−84.14 ± 15.82	−484.09 ± 101.77

**Table 4:** Molecular interaction energy profile of αM–Kollidon^®^ VA64 systems from MM/PBSA calculations.

Ratio (αM:Polymer)	Van der Waals Energy (kJ/mol)	Electrostatic Energy (kJ/mol)	Polar Solvation Energy (kJ/mol)	SASA Energy (kJ/mol)	Binding Energy (kJ/mol)
1:1	−75.07 ± 41.61	−7.55 ± 10.90	24.42 ± 21.47	−13.38 ± 6.64	−71.58 ± 40.28
1:3	−235.96 ± 72.76	−23.91 ± 18.60	89.76 ± 37.07	−40.73 ± 10.92	−210.83 ± 67.59
1:5	−345.55 ± 87.71	−38.56 ± 22.60	139.63 ± 42.33	−58.37 ± 12.46	−302.84 ± 84.87
1:7	−427.05 ± 95.44	−49.08 ± 26.45	165.20 ± 48.73	−71.48 ± 13.56	−382.41 ± 86.58

**Table 5:** Calculated composition ratios and molecular counts of αM–Soluplus^®^ amorphous solid dispersion systems.

Ratio (α-Mangostin:Polymer)	Scaling Factor (K)	Number of Simplified Structural Units	
		α-Mangostin	Soluplus^®^
1:1	1.50	8	12
1:3	4.50	8	36
1:5	7.50	8	60
1:7	10.75	8	86

**Table 6:** Calculated composition ratios and molecular counts of αM–Kollidon® VA64 amorphous solid dispersion systems.

Ratio (α-Mangostin:Polymer)	Scaling Factor (K)	Number of Simplified Structural Units	
		α-Mangostin	Kollidon^®^ VA64
1:1	2.00	8	16
1:3	6.25	8	50
1:5	10.50	8	84
1:7	14.50	8	116

## Data Availability

The data generated and analyzed during this study are available from the corresponding author upon reasonable request. The data include the molecular structures, topology, and molecular dynamics parameter files, processed trajectory data, and numerical datasets supporting the RMSD, RMSF, RDF, radius of gyration, solvent-accessible surface area, hydrogen-bond, and MM/PBSA analyses.

## Supplementary Materials

The following supporting information can be downloaded at: https://www.mdpi.com/article/10.3390/ijms27167283/s1.
