# Supplementary material for: Molecular Dynamics Simulation and MM/PBSA Analysis of α-Mangostin Stabilization in Soluplus® and Kollidon® VA64-Based Amorphous Solid Dispersions
Source: Int J Mol Sci. 2026 Aug 15;27(16):7283. doi: 10.3390/ijms27167283 (PMC13513123; doi:10.3390/ijms27167283)
Supplement: Supplementary file 1 [file ijms-27-07283-s001.zip › ijms-4425875-supplementary.pdf]

Supplementary Document

Table S1. Molecular composition and calculation of the simplified computational structural units used to construct the α-mangostin–polymer systems

**Component**	**Representation in the computational model**	**Constituent structural unit**	**Molecular formula**	**Molecular weight (g/mol)**	**Reciprocal molecular weight (mol/g)**
α-Mangostin	Complete α-mangostin molecule	α-Mangostin	$C_{24}H_{26}O_6$	410.46	0.002436
Soluplus®	One simplified composite structural unit	N-Vinylcaprolactam (VCL)	$C_8H_{13}NO$	139.20	—
		Vinyl acetate (VAc)	$C_4H_6O_2$	86.09	—
		Ethylene oxide (EO)	C_2H_4O	44.05	—
	Combined representative Soluplus® unit	1 VCL + 1 VAc + 1 EO	$C_{14}H_{23}NO_4$	269.34	0.003713
Kollidon® VA64	One simplified composite structural unit	N-Vinyl-2-pyrrolidone (VP)	C_6H_9NO	111.14	—
		Vinyl acetate (VAc)	$C_4H_6O_2$	86.09	—
	Combined representative Kollidon® VA64 unit	1 VP + 1 VAc	$C_{10}H_{15}NO_3$	197.23	0.005070
